# Metabolic costs of bat echolocation in a non-foraging context support a role in communication

**DOI:** 10.3389/fphys.2013.00066

**Published:** 2013-04-04

**Authors:** Dina K. N. Dechmann, Martin Wikelski, Hendrika J. van Noordwijk, Christian C. Voigt, Silke L. Voigt-Heucke

**Affiliations:** ^1^Department of Migration and Immuno-Ecology, Max Planck Institute for OrnithologyRadolfzell, Germany; ^2^Department of Biology, University of KonstanzKonstanz, Germany; ^3^Department of Evolutionary Ecology, Leibniz Institute for Zoo and Wildlife ResearchBerlin, Germany; ^4^Department of Animal Behavior, Institute of Biology, Freie Universität BerlinBerlin, Germany

**Keywords:** Chiroptera, energetic costs, *Noctilio albiventris*, cue, signal, fitness

## Abstract

The exploitation of information is a key adaptive behavior of social animals, and many animals produce costly signals to communicate with conspecifics. In contrast, bats produce ultrasound for auto-communication, i.e., they emit ultrasound calls and behave in response to the received echo. However, ultrasound echolocation calls produced by non-flying bats looking for food are energetically costly. Thus, if they are produced in a non-foraging or navigational context this indicates an energetic investment, which must be motivated by something. We quantified the costs of the production of such calls, in stationary, non-foraging lesser bulldog bats (*Noctilio albiventris*) and found metabolic rates to increase by 0.021 ± 0.001 J/pulse (mean ± standard error). From this, we estimated the metabolic rates of *N. albiventris* when responding with ultrasound echolocation calls to playbacks of echolocation calls from familiar and unfamiliar conspecific as well as heterospecific bats. Lesser bulldog bats adjusted their energetic investment to the social information contained in the presented playback. Our results are consistent with the hypothesis that in addition to orientation and foraging, ultrasound calls in bats may also have function for active communication.

## Introduction

Information use has been proposed as key adaptive behavior (Danchin, 2004), with communication systems arising when it is important for two individuals to intentionally exchange this information to the benefit of both (Seyfarth and Cheney, 2003; Seyfarth et al., 2010). Animals can intentionally transmit information in the form of “signals” and the resulting active communication should be the core mediator of animal interactions. However, information between animals can also be transmitted via inadvertently produced “cues” which can alter the behavior of an active receiver as well. Cues and signals share several key features: a communicator or sender, information (signal or cue), and a recipient (Danchin, 2004). There is therefore a major distinction between inadvertent cues and intentional signaling and how selection can act on both. According to Maynard-Smith and Harper (2003) a signal is “any act which alters the behavior of other organisms that has evolved because of that fact, and which is effective because of the receiver's response that has also evolved.” This requirement that a signal evolved due to its effect on other organisms, is a fundamental difference from cues, which are simply by-products of the producer's action and not under selection for information transfer from either the sender's or receivers viewpoint (Scott-Phillips, 2008).

The long-term currency of communication is Darwinian fitness; the short-term currency is energy or time expended by a sender. If communication is taking place at all, maximizing fitness forces animals to optimize communication and thus selection acts on both, signals (the sender and receiver side) and cues (only on the receiver side). Senders of signals invest energetic costs or time, if the cost to maintain such a signal plays a major role in securing the information content, i.e., the honesty of a signal (Zahavi, 1975, 1977), whereas the receiver can invest considerable energy, time, and predation risks to receive and process both cues and signals and may have to adapt to this in the course of evolution (Bradbury and Vehrencamp, 2011). The role of signals and cues and how the latter may turn into the former is very context-dependent and closely tied to the modality in which they are produced (e.g., sound, vision, olfaction).

One group of animals that constantly and involuntarily produce auditory cues while moving are echolocating bats. Echolocation calls are vocalizations, usually in the ultrasound range above 20 kHz, enabling bats to orientate and forage at night. Echolocation has been described as “autocommunication” (Bradbury and Vehrencamp, 2011) or “communication about the environment surrounding oneself” (Simmons, 1977), with the same bat operating as both signaler and receiver. Echolocation is under strong selection, because call structure, frequency, and intensity are largely determined by the type of prey, amount of background clutter and phylogenetic history of species. Thus, it has been assumed that there would be little or no adaptive plasticity allowing additional communicative information to be contained in them (Schnitzler et al., 2003), although the idea of communicative elements being contained in them is not new (Möhres, 1967). Recently, evidence has been accumulating that bats also act and react in the presence of and in response to other echolocating bats (reviewed in Jones and Siemers, 2010). Echolocation may in fact have evolved from social vocalizations (Fenton, 1984), and the potential for communication may be much higher than previously assumed.

Metabolic costs of acoustic signaling are about eight times that of the silent animal in several taxa (Ophir et al., 2010), and the cost of echolocation can be even higher. For example, in an experimental situation, a stationary 6 g pipistrelle bat looking for food, but adapted to foraging for insects on the wing, spends approximately 0.067 J/pulse (Speakman, 1989) and a 17 g *Eptesicus fuscus* metabolizes 0.197 J/pulse (Speakman et al., 2004). It is unknown how much stationary echolocation is used in a natural scenario, but this would add up to 1.3 and 3.9 J/s, respectively, at up to 20 calls per second, very high compared to *E. fuscus*' daily energy expenditure of about 30 kJ (Kurta et al., 1989, 1990). In contrast, there seems to be no additional cost of echolocation in flying bats (Speakman, 1991), and one should assume that stationary bats should rarely echolocate especially when not trying to locate food. We do not know how much spontaneous echolocation is used in stationary bats under natural conditions, but if bats do intentionally produce these energetically costly calls in non-foraging contexts, instead of active information transfer, this might indicate that these calls not only serve as cues, but might be energetically costly signals in bat communication.

### Cost of echolocation in bat communication—a case study of *Noctilio albiventris*

*Noctilio albiventris* is a Neotropical bat species that roosts in groups of 5–20 individuals in our study area, Gamboa, Panama (09.078N°; 079.418°W). Radio-telemetry data revealed that group members coordinate flight to forage together (Dechmann et al., 2009), and playback experiments demonstrated that this allows eavesdropping on inadvertent information contained in “feeding buzzes,” calls produced during attempts to capture prey (Dechmann et al., 2009). A sudden increase in feeding buzzes is a cue that indicates a profitable feeding patch to eavesdropping group members. These results showed non-opportunistic use of cues in a social context, a behavior otherwise only described in dolphins (Lammers and Au, 2003; Lammers et al., 2003). An additional set of playback experiments with captive *N. albiventris* indicated that echolocation calls may be used not only for eavesdropping on the wing, but also in an exclusively social context (Voigt-Heucke et al., 2010). Stationary, non-foraging *N. albiventris* responded with more social behaviors including more echolocation calls to playbacks of orientation echolocation calls of their own species than those of other species, and even more intensively to calls of unfamiliar conspecifics than individuals of their own social group. This was surprising because due to the high energetic costs of producing ultrasound echolocation calls (in contrast to social calls, which may also be produced in the ultrasound range), non-flying bats were not expected to echolocate more than necessary for orientation or localizing food. In addition, the cue used for eavesdropping by foraging bats are feeding buzzes and not the orientation calls used in the experiment described above.

To investigate if echolocation may also serve as an active signal in bat communication, we first measured the energetic costs of echolocation in non-foraging, non-flying bats. We then used the data published in Voigt-Heucke et al. (2010) to quantify the investment in different social contexts. If echolocation serves a communicative function, we expected metabolic rates to increase significantly in *N. albiventris* producing ultrasound calls in response to the calls of another bat.

## Methods

### Energetic cost of echolocation in *Noctilio albiventris*

We caught adult *Noctilio albiventris* between 7 and 9 pm during March 2009 and 2010 with mistnets (Ecotone, Poland) when they were returning with full bellies to known daytime roosts after foraging in the vicinity of the village Gamboa, Panama. After determining sex, age, reproductive state, and forearm length in mm with calipers (Mahr, Germany) and weighing them to the nearest 0.25 g (Pesola, Switzerland), bats were transferred to a nearby laboratory in soft cloth bags. There, they were placed in the metabolic chamber (1l volume) of a respirometry setup (see below). The metabolic chamber, which was lined with wire mesh, allowed the animals to roost on the side of the container in a natural position, but did not allow them to fly. The chamber was padded with rubber foam to avoid reflections of echolocation calls. An infrared-sensitive video camera (Sony, Japan) confirmed that calling bats were not moving except for turns of the head. The measurements were made in a dark silent room at ambient humidity and temperature (25°C) and the bat's calling activity was monitored and recorded from outside the room on a computer screen. To record echolocation calls, we placed an Avisoft condenser ultrasound microphone CM16/CMPA (Avisoft Bioacoustics, Germany) near the bat's head. The microphone was connected to an Avisoft UltraSoundGate 116Hme, which directly recorded onto a laptop computer with the Avisoft software Recorder USGH version 3.4. Recordings were performed with a 16 bit resolution and a 250 kHz sampling rate.

Some bats remained silent for up to 1 h after being placed in the chamber and all remained silent at least 10 min. And many bats never spontaneously called at all. We measured resting (i.e., immobile and not calling) and calling (i.e., echolocating, but not moving more than the head) metabolic rate and released the bats after they had vocalized (*n* = 7; mass: 25.8 ± 3.2 g; six females, one male). Bats that had not vocalized after 1 h (*n* = 2) were released without recording. All bats were released at the daytime roost before midnight of the capture night.

We measured the oxygen consumption and carbon dioxide production of calling and silent *N. albiventris* using an open-flow, push-through respirometry system. Ambient air with a humidity of about 85–95% was pumped at a flow rate of 1 lmin^−1^ via a mass flow controller (TR-FCI, Sable Systems, Las Vegas, NV) and a multiplexer (V2-0, Sable Systems) into the chamber. Reference values were taken before and after the animals were placed in the chamber. After dehumidifying inlet air with a Peltier-Effect Condenser (PC-1, Sable Systems), we measured CO_2_ concentration from a sub-sample (CA 1B, Sable Systems). We used drierite to scrub off potential remaining water from the air, and then measured oxygen concentration.

We used the equation by Bartholomew and co-authors (1981) to measure instantaneous oxygen consumption rate

$$FE_{o_{2}}(eq)=FE_{o_{2}}(t-1)+\left[\frac{FE_{o_{2}}(t)-FE_{o_{2}}(t-1)}{1-e^{(-v>>/v)\Delta t}}\right]$$

where *FE*_*o*2_ is the oxygen consumption in the outlet air, *FE*_*o*2_ (eq) is the equilibrium value, V is the volume of the respirometry system, *v* >> is the flow rate through the system, and Δt is the interval between measurements at times *t* and *t*-1. The denominator of the equation was determined empirically with Datacan (Sable Systems). The rate of oxygen consumption was calculated using Equation (3b) of Withers (1977).

We converted oxygen consumption rate into energy turnover by utilizing the caloric equivalent of protein oxidation (Voigt et al., 2010). After having fed on their insect diet Noctilio used proteins as a metabolic fuel (Voigt et al., 2010). The caloric equivalent for endogenous carbohydrate or fat oxidation is almost the same: 19.6 kJ/lO2 for fat oxidation, 21.1 kJ/lO2 for carbohydrate oxidation (and 18.8 Kj/lO2 for protein oxidation; Penzlin, 1989). The acoustical recordings were started simultaneously with the measurements of oxygen consumption. We counted the number of calls per second throughout metabolic measurements. As the microphone was at a distance of only 5 cm to the bat's head, call intensity very much depended on the orientation of the animal's head, Consequently, it was not possible to quantify sound pressure levels.

Oxygen consumption was not measured with the same animals that were used by Voigt-Heucke and coauthors to assess the behavioral responses to echolocation playbacks (see below). Thus, a mean cost per echolocation call was calculated based on our data.

### Call rate in response to different social contexts (all data from Voigt-Heucke et al., 2010)

All data cited in this paragraph are from the cited study, for more details on behavioral response data collection to different playbacks, including animal housing, preparation of the playback files etc. see the original paper (Voigt-Heucke et al., 2010). In summary: four types of playback stimuli were used to quantify the reaction of *N. albiventris* to echolocation calls in a social context. Stimulus categories were orientation calls from (1) familiar conspecifics (group members, *n* = 15 individuals from three social groups), (2) unfamiliar conspecifics (non-group members, *n* = 5), (3) heterospecifics that share roosts with *N. albiventris* (*Molossus molossus*, *n* = 5), and (4) heterospecifics that do not share roosts (*Uroderma bilobatum*, *n* = 5). Here, the experimental animals were 20 experimentally naïve individuals from the three “familiar conspecifics” groups. Bats were allowed to habituate to the experimental situation for at least 30 min before the start of experiments. The bats' behavioral response to the playback was then filmed and their acoustic response recorded. Each bat was tested in five trials. Stimulus categories were presented in random order during these five trial sessions, and only one trial was conducted per night with each bat to avoid habituation.

Each playback trial consisted of three phases: a pre-playback phase (2 min), a playback phase (8 s) and a post-playback phase (5 min). The pre-playback phase started when bats had been hanging motionless and silent for at least 2 min. For analysis the echolocation response rates (n/5 min) of each bat during the 5 min post-playback phase was assessed.

### Statistical analysis—cost of echolocation in a social context

To describe the relationship between call rate (*n*/10 s) and oxygen consumption we built a generalized linear mixed model in *lme4* (Bates et al., 2011). We included call rate as a fixed factor and individual as random intercept factor to correct for differences between individuals. As they bats were caught after foraging (which can add about one third to their mass), and the estimations were done with oxygen consumption of one, but response rates of another set of individuals, we corrected for individual and did not additionally include mass after running a simulation with mass that did not affect the results. We ran a model with random slope and intercept and compared it to the random intercept only model (with full fixed factors) using REML estimation as suggested by Schielzeth and Forstmeier (2009). We then bootstrapped the model 10,000 times using the *arm* package to obtain the distribution of the likelihood ratio (Gelman et al., 2011). As the random slope model was no better than the random intercept only model we estimated the fixed effects with a random intercept only model and ML for the estimates. *P*-values for the fixed effects were estimated with a Markov Chain Monte Carlo (MCMC) approach in *languageR* (Baayen, 2011). All analyses were performed in R 2.13 (R-Development-Core-Team, 2009).

We used the equivalent of oxygen consumption per echolocation pulse (assessed by us), to extrapolate the response costs of 20 *N. albiventris* from Voigt-Heucke et al. (2010). We log(x + 10)-transformed the response costs and calculated a repeated measures analysis of variance to test for difference in response costs (number of calls/10 s) to the stimuli “familiar (FC)” and “unfamiliar conspecifics (UC),” and “cohabitant (CH)” and “non-cohabitant heterospecifics (NCH).” *Post-hoc* Tukey–Kramer tests were used for pair-wise differences between the energy costs of stimuli responses. All tests were two-tailed with an assumed alpha value of 5%. Data are presented as mean ± one standard deviation if not otherwise stated.

## Results

### Energetic costs of echolocation in *Noctilio albiventris*

**Figure 1:**
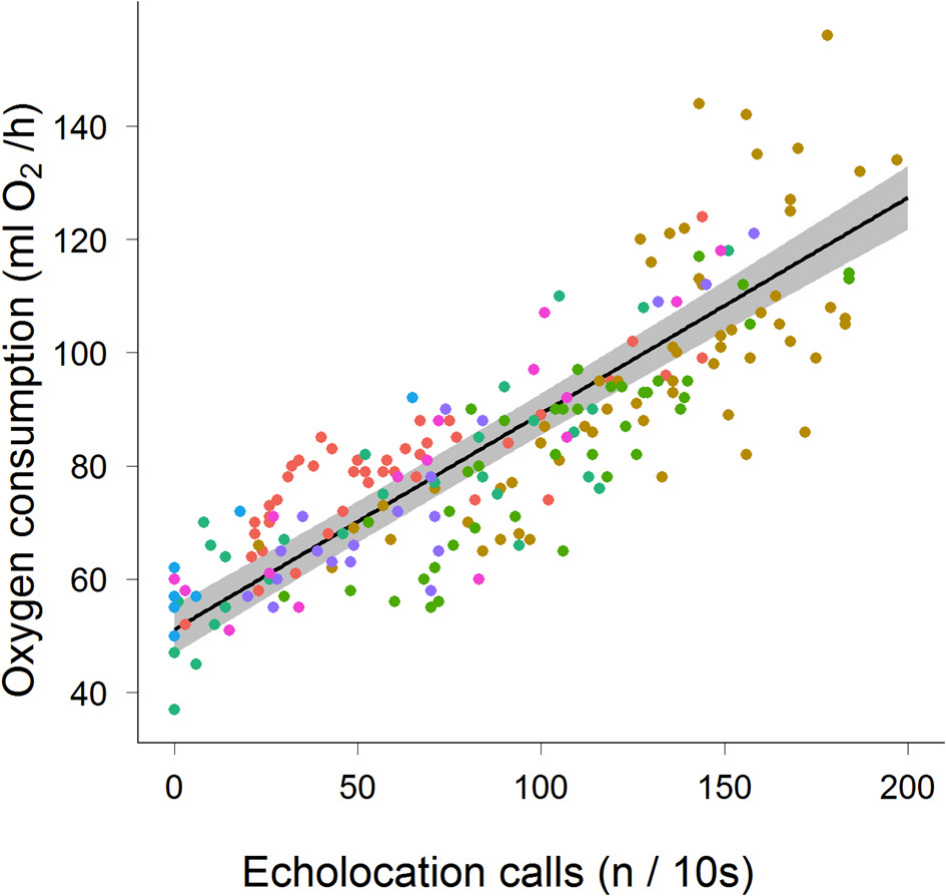
**Metabolic rate (ml O_2_/h) for seven *Noctilio albiventris* (indicated by different colors) in relation to echolocation pulse rate (n/10 s).** The mean metabolic rate as estimated by the model is the black line with the 95% credible interval indicated by the gray area.

We recorded the energy consumption of seven bats calling during bouts lasting 18–586 s that produced a maximum of 29 calls/s. The mean (±stdev) metabolic cost of non-calling non-foraging, stationary bats was 51.9 ± 5.7 ml O_2_ h^−1^. When calling costs ranged between 50 and more than 120 ml O_2_ h^−1^ (Figure 1, Table 1). We found a significant positive relationship between call rate and oxygen consumption (Figure 1): rate of oxygen consumption (ml O_2_ h^−1^) = 51.13 + 0.38 × call rate (n/10 s).

**Table 1:** **Model estimates of the relationship between rate of oxygen consumption (ml O_2_/h) and call rate (n/10 s)**.

	**Estimate ± std error**	***t* value**	**Lower 95%**	**Upper 95%**	**pMCMC**
Intercept	51.14 ± 2.25	22.75	46.15	56.26	0.0001
Call rate	0.38 ± 0.018	21.79	0.35	0.42	0.0001

pMCMC, probability calculated with Markov Chain Monte Carlo approach.

### Cost of echolocation in a social context

Calculating oxygen consumption at the pulse rates the bats responded to the social playbacks with, using the equation from the mixed model showed that the bats adjusted their response depending on the presented stimulus [*F*_(3, 57)_ = 3.48; *P* = 0.0257; Table 2]. The energetic costs of responses were significantly higher when exposed to unfamiliar conspecifics than to both types of heterospecifics (Table 2).

**Table 2:** **Pair-wise comparisons using Tukey–Kramer *post-hoc* tests**.

**comparison**	**q**	***p*-Value**
FC-UC	2.41	n.s.
FC-CH	1.46	n.s.
FC-NCH	1.45	n.s.
UC-CH	3.88	<0.05
UC-NCH	3.86	<0.05
CH-NCH	0.18	n.s.

FC, familiar conspecific; UC, unfamiliar conspecific; CH, cohabitant heterospecific; NCH, non-cohabitant heterospecifc; q, Tukey-Kramer statistic; n.s., non-significant. Data from Voigt-Heucke et al. (2010).

## Discussion

We estimated the metabolic costs of the spontaneous ultrasound response in non-foraging, stationary bats, in response to different social stimuli, and showed that echolocation in *N. albiventris* incurs substantial energy costs. Calling bats spent 0.0213 J/pulse, about 2–5 times more energy than silent bats. This is lower than previously recorded for other species, most likely due to larger size of Noctilio, but still substantially higher than non-calling energy expenditure. Bats increase call rates significantly in response to playbacks of unfamiliar conspecifics (Voigt-Heucke et al., 2010) and thus adjust their energetic investment according to the social information perceived in the presented playback, an indicator of active signaling. Jamming avoidance or increased call rates to improve foraging efficiency in a competitive situation, cannot explain the bats' costly response in an experimental situation. Female mice are more interested in the odors of unknown than of known males (Kavaliers, 2003) and, similarly, bats respond more strongly to the calls of unfamiliar conspecifics than heterospecifics (Voigt-Heucke et al., 2010) which translates into significantly higher costs. The exact purpose of this remains unknown, but several non-exclusive interpretations are possible. The calls may convey information about the sender's identity, sex, or quality, but they may also be a dominance, aggression, or appeasement gesture.

Most animals produce sounds specifically for communication, a typical example being bird song. In contrast, bat echolocation calls are primarily produced for orientation and foraging and are under strong selection for adaptation to this niche specific purpose. Nonetheless evidence has been accumulating that the communicative potential of bat echolocation is high [reviewed in Jones and Siemers (2010), Knörnschild et al. (2012)]. The ultrasound calls of bats, which are adapted to foraging on the wing are very costly when the bats are presented with food in a stationary situation (Speakman, 1989; Speakman et al., 2004). In contrast, calls emitted on the wing during foraging and orientation are not costly (Speakman, 1991) probably due to the timing of call emission with the wing beat upstroke and exploiting the power generated by the resulting muscle contractions. All studies that try to determine the communicative function of echolocation, including our own, have used the number of echolocation calls as response variable (Kazial, 2004, 2008; Yovel et al., 2009; Schuchmann and Siemers, 2010; Voigt-Heucke et al., 2010; Schuchmann et al., 2012). An increase in call rates on the wing could be interpreted as an attempt to be more competitive in a foraging situation, and flying *Noctilio albiventris* in the field do indeed react to playbacks of feeding buzzes of unfamiliar individuals by approaching them (Dechmann et al., 2009; Übernickel et al., 2012). In the proper experimental context, changes in echolocation rates could even be interpreted as an intentionally produced vocalization with the goal to alter the behavior of the caller or to indicate, for example, individual identity, sex, group membership, or species, fulfilling at least one of the conditions for the definition of a signal as proposed by Maynard-Smith and Harper (2003), however, this had not been tested before.

The amount of time bats during our measurements of oxygen consumption spent echolocating varied from just two short bouts of less than 10 s (a female) to 50 or more 10 s bouts (also a female). Our sample of seven bats was composed of a male and pregnant as well as non-reproductive females, showing a high correlation between call rate and oxygen consumption, which gives us confidence that these data are a good enough representation of the species' behavior and energy investment to indicate that an investment is in fact being made.

Based on our results, we advocate that depending on the context, echolocation calls may either be used as cues produced by foraging conspecifics, i.e., eavesdropping on feeding buzzes; or intentionally produced costly signals. Whereas it has often been shown that signals, such as mating calls can also serve as cues for other con- and heterospecifics, our data are consistent with the hypothesis that even though echolocation calls are mainly strongly selected autocommunicative signals in an ecological context, they may in addition be actively produced signals for social communication.

### Conflict of interest statement

The authors declare that the research was conducted in the absence of any commercial or financial relationships that could be construed as a potential conflict of interest.
